# Effects of an Occupation-Based Group Occupational Therapy Intervention on Cognitive Functioning in Adults with Schizophrenia: A Randomized Controlled Trial

**DOI:** 10.3390/neurolint18100182

**Published:** 2026-09-25

**Authors:** Elpida Stratou, Christina Athanasopoulou, Aristotelis Koinis, Georgia-Nektaria Porfyri, Symeon Dimitrios Daskalou, Nikolaos Gerosideris, Evangelia Arvanitidou, Aikaterini Gamvroula, George Pierrakos, Ioanna Giannoula Katsouri

**Affiliations:** 1 Department of Occupational Therapy, University of West Attica, 12243 Athens, Greece; chatha@uniwa.gr (C.A.); sdaskalou@uniwa.gr (S.D.D.); ngerosideris@uniwa.gr (N.G.); earvanitidou@uniwa.gr (E.A.); ykatsouri@uniwa.gr (I.G.K.); 2 Department of Psychiatry, General Hospital of Argolida, 21231 Argos, Greece; geoporfyri@hotmail.fr (G.-N.P.); gamvroulak@gmail.com (A.G.); 3 Department of Nursing, University of Thessaly, 41500 Larissa, Greece; aristokoinis@uth.gr; 4 Department of Business Administration, University of West Attica, 12244 Athens, Greece; gpierrakos@uniwa.gr

**Keywords:** schizophrenia, occupational therapy, occupation-based intervention, cognitive impairment, cognitive test performance, group intervention, randomized controlled trial

## Abstract

**Background/Objectives:** Cognitive impairment is a core feature of schizophrenia and is closely associated with everyday functioning and participation. This study examined the effects of a 12-week occupation-based group occupational therapy intervention on cognitive functioning in adults with schizophrenia. **Methods:** In this two-arm randomized controlled trial, 86 adults with schizophrenia were randomly allocated to an intervention group (*n* = 44) or a treatment-as-usual control group (*n* = 42). The intervention combined cognitive activation with everyday tasks, problem-solving, and social and creative activities. Co-primary outcomes were the Mini-Mental State Examination (MMSE), Addenbrooke’s Cognitive Examination III (ACE-III) total score, and Clock Drawing Test (CDT). Secondary outcomes included the Mini-Addenbrooke’s Cognitive Examination (M-ACE), ACE-III domains, and Trail Making Test (TMT) Parts A and B completion times and error counts, assessed at baseline and post-intervention. Group × time effects were examined using repeated-measures analysis of variance. **Results:** All 86 participants completed the post-intervention assessment and were included in the analysis. Compared with treatment as usual alone, the intervention was associated with significantly greater improvements in MMSE, ACE-III total, M-ACE, TMT Part A completion time, and the ACE-III language and visuospatial domains. Partial η^2^ values ranged from 0.17 to 0.43, with the largest effect observed for the ACE-III total score. **Conclusions:** Adding occupation-based group occupational therapy to usual care may improve global and domain-specific cognitive test performance in adults with schizophrenia. Further research should determine whether these changes are maintained and translate into everyday occupational performance and participation. This trial was retrospectively registered with ISRCTN (ISRCTN43725423).

## 1. Introduction

Schizophrenia is a severe mental disorder characterized by positive and negative symptoms and substantial disturbances in social and occupational functioning [1]. Cognitive impairment is also widely recognized as a core feature of the disorder and is closely associated with poorer everyday and independent functioning and reduced quality of life [2,3]. Frequently affected cognitive domains include processing speed, attention, working memory, verbal and visual learning and memory, reasoning and problem-solving, and executive functioning [3,4].

The functional relevance of cognitive impairment extends beyond performance on neuropsychological tests, as both neurocognition and social cognition are consistently associated with real-world and community functioning in schizophrenia [5,6]. Difficulties in attention, memory, processing speed, and executive functioning may limit individuals’ ability to organize daily routines, manage complex everyday activities, live independently, and engage in occupational and social roles [2,5]. These relationships highlight the importance of systematically assessing cognitive functioning and addressing it as a treatment and rehabilitation target alongside the management of clinical symptoms [3,7,8].

Current evidence supports a comprehensive approach to schizophrenia care, in which pharmacological treatment is complemented by evidence-based psychosocial and rehabilitation interventions addressing clinical symptoms as well as cognitive and functional outcomes [4,9,10]. Among these interventions, cognitive remediation has demonstrated beneficial effects on cognition and functioning, particularly when it incorporates structured cognitive strategy development and is integrated into psychosocial rehabilitation [11,12].

According to the Occupational Therapy Practice Framework: Domain and Process, occupational therapy aims to promote health, well-being, and participation in life through engagement in occupation [13]. Occupational performance is considered in relation to occupations, contexts, performance patterns, performance skills, and client factors [13]. Applied to cognition, this perspective draws attention not only to specific cognitive abilities but also to how these abilities are used during the performance of everyday tasks, routines, and roles [13,14]. The concept of functional cognition reflects this perspective, referring to the integration and use of cognitive and performance abilities in everyday life as a fundamental component of occupational performance [14].

Building on this perspective, occupation-based interventions may provide structured opportunities for individuals with schizophrenia to apply cognitive strategies while performing everyday tasks, managing routines, solving practical problems, and engaging in social and community activities. Rather than addressing cognitive processes exclusively through isolated exercises, such interventions seek to support the application of cognitive abilities within meaningful everyday occupations [13,14]. Recent evidence, including a multicenter randomized controlled trial and a systematic review, suggests that occupational therapy interventions may improve several cognitive outcomes in schizophrenia [15,16]. However, evidence remains limited and heterogeneous, and relatively little is known about the effects of structured, occupation-based group interventions delivered within routine clinical settings [16].

Although the present intervention incorporated elements that may also be encountered in cognitive remediation, including structured cognitive practice, strategy use, and problem-solving [11,12], its defining feature was the use of occupation as the primary therapeutic medium. The programme was organized around engagement in meaningful occupations and occupation-based activities involving daily routines, real-world problem-solving, social and community participation, and creative expression. Within this framework, cognitive activation exercises functioned as preparatory or supportive components and were consistently connected to broader occupational themes and their potential application in everyday life, rather than delivered as a self-contained cognitive training programme. This occupation-based organization conceptually and practically differentiates the intervention from a conventional cognitive remediation format. Its novelty lies in the structured integration of cognitive demands, strategy use, and reflection within meaningful group occupations delivered in a routine psychiatric service [13,14,16].

Accordingly, the present randomized controlled trial aimed to examine the effects of a 12-week occupation-based group occupational therapy intervention on cognitive test performance in adults with schizophrenia. The intervention integrated cognitive activation, everyday task practice, functional problem-solving, social participation, and creative expression, thereby embedding cognitive demands within meaningful and structured occupations and occupation-based activities. It was hypothesized that participants receiving the intervention in addition to treatment as usual would demonstrate greater improvements from baseline to post-intervention in global and domain-specific cognitive test performance than those receiving treatment as usual alone.

## 2. Materials and Methods

### 2.1. Study Design

This study was designed as a 12-week, two-arm, parallel-group randomized controlled trial. Participants were randomly allocated with an intended 1:1 allocation ratio to either the intervention group, which received treatment as usual in addition to a structured, occupation-based group occupational therapy intervention, or the control group, which received treatment as usual alone. Assessments were conducted at baseline (T0) and immediately after completion of the 12-week intervention (T1), using the same cognitive assessment instruments at both time points.

The main aim of the study was to evaluate the effects of the intervention on cognitive test performance in adults with schizophrenia, including global cognitive functioning, attention, memory, verbal fluency, processing speed, visuospatial abilities, and executive functioning. The ACE-III total score, MMSE total score, and CDT score were designated as the co-primary cognitive outcomes. Secondary cognitive outcomes included the M-ACE score, ACE-III domain scores, and TMT Parts A and B completion times and error counts.

The broader trial also assessed psychopathology, psychosocial functioning, health-related quality of life, and satisfaction with mental health services using the PANSS, GAF, SF-36, and VSQ-9, respectively. The cognitive outcome domain was established in the original study protocol before participant enrolment and constitutes the focus of the present report. To provide complete reporting of the registered secondary outcome domains, longitudinal PANSS, GAF, SF-36, and VSQ-9 outcomes are reported in Supplementary Tables S1 and S2 and summarized briefly in the Results. Baseline PANSS scores are also reported descriptively to characterize the baseline psychopathology profile of the two groups. This report was prepared in accordance with the CONSORT 2025 statement and the CONSORT extension for randomized trials of nonpharmacologic treatments [17,18].

The study was conducted in accordance with the Declaration of Helsinki. Ethical approval was obtained from the Research Ethics Committee of the University of West Attica on 31 January 2024 (approval no. 5765/31-01-2024). The study was also approved by the Scientific Council of the General Hospital of Argolida on 9 June 2023 (approval no. 11414/09-06-2023) and authorized by the 6th Regional Health Authority of Peloponnese, Ionian Islands, Epirus and Western Greece on 12 May 2023 (authorization no. 28008/12-05-2023). All approvals were obtained before participant enrolment and data collection. Written informed consent was obtained from all participants before enrolment. The trial was retrospectively registered with the ISRCTN registry on 10 July 2026, following study completion (ISRCTN43725423). The study was undertaken as an investigator-led academic research project under prior institutional and ethical approval; however, prospective trial registration was not completed before participant enrolment due to a misunderstanding regarding the applicability of the prospective registration requirement to non-pharmacological intervention trials. The trial was subsequently registered to ensure public availability and transparency of the trial information. The core study design, intervention schedule, assessment time points, and principal outcome domains had been defined before participant enrolment.

### 2.2. Participants

Participants were adults with a diagnosis of schizophrenia recruited from the Psychiatric Department of the General Hospital of Argolida-Nursing Unit of Argos, Greece, including the depot medication clinic, outpatient psychiatric clinics, and the Mobile Mental Health Unit. The diagnosis of schizophrenia was documented in participants’ medical records according to ICD-10 criteria (F20) and had been confirmed by their treating psychiatrists. All participants had been under continuous psychiatric follow-up for at least one year before enrolment.

Eligibility criteria included age between 18 and 70 years; a documented diagnosis of schizophrenia; clinical stability for at least six months before enrolment, as confirmed by the treating psychiatrist on the basis of clinical evaluation and the absence of an active psychotic episode or severe clinical relapse; no prespecified PANSS cutoff was used as an eligibility criterion; sufficient cognitive and communication ability to understand the assessment instructions and participate in group occupational therapy sessions; ability to provide written informed consent independently; and no concurrent participation in another structured therapeutic or psychosocial programme during the study period. Treatment as usual consisted of ongoing psychiatric follow-up and prescribed pharmacological treatment provided through the routine services of the Psychiatric Department. Participants in both groups remained on their prescribed pharmacological treatment throughout the 12-week study period, with no changes in medication type or dosage documented during the study period.

Exclusion criteria included a history of a neurological or neurodevelopmental disorder; intellectual disability or severe cognitive impairment not attributable to schizophrenia; a history of severe traumatic brain injury involving loss of consciousness; active alcohol or substance abuse or dependence; an active psychotic episode or severe clinical relapse at the time of recruitment; and inability to provide informed consent independently, including individuals whose legal status required consent through a court-appointed representative.

The original study protocol anticipated a sample of 60 participants. Before participant enrolment, the required sample size was reassessed through an a priori power analysis conducted using G*Power version 3.1 [19] for a repeated-measures ANOVA (within-between interaction) design, with the ACE-III total score, MMSE total score, and Clock Drawing Test (CDT) score designated as the co-primary cognitive outcomes. The analysis was based on detecting a group with time interaction corresponding to a conservative effect size of f = 0.20 (equivalent nearly to a partial η^2^ = 0.038), with an alpha level of 0.05, a statistical power of 95%, two study groups, two measurement time points, and an assumed correlation among repeated measures of 0.50. This calculation indicated a required total sample size of 80 participants (40 per group). To account for potential loss to follow-up, the recruitment target was increased to 90 participants, with an intended allocation of 45 participants per group. Eligibility was confirmed through a review of medical records, a clinical interview, and consultation with the treating psychiatrists before enrolment. A total of 90 individuals were assessed for eligibility, of whom four were excluded before randomization; consequently, 86 participants were enrolled and randomized. Further details regarding participant recruitment and allocation are presented in the CONSORT flow diagram (Figure 1). Patients and members of the public were not involved in the design, conduct, reporting, or dissemination planning of the trial.

### 2.3. Randomization

Participants were randomly allocated to the intervention or control group using complete randomization with a prespecified 1:1 allocation ratio, without blocking or stratification. Before participant enrolment commenced, an independent healthcare professional created 90 allocation entries, comprising 45 assignments to each group. Each entry was assigned a random number using the RAND() function in Microsoft Excel, and the entries were sorted according to the generated random numbers to produce the allocation sequence. Because 86 participants were ultimately enrolled and randomized, the first 86 assignments were used, resulting in 44 participants allocated to the intervention group and 42 to the control group. The independent healthcare professional was not involved in participant assessment or delivery of the occupational therapy intervention.

Allocation concealment was ensured using sequentially numbered, opaque, sealed envelopes containing the group assignments and prepared by the same independent healthcare professional. The electronic allocation list was retained by the independent healthcare professional and was not accessible to the investigator responsible for participant enrolment. The envelopes were signed across the sealed flap to provide evidence of any prior opening, and the allocation sequence remained concealed from the investigator until the corresponding envelope was opened. After eligibility had been confirmed and written informed consent had been obtained, the next envelope in numerical order was opened by the investigator, and the indicated allocation was implemented without modification. Participants were assigned unique numerical codes, which were used on the assessment forms to protect confidentiality. Due to the nature of the intervention, participants and the occupational therapist delivering the intervention could not be blinded to group allocation. Outcome assessors were also aware of participants’ group allocation. Independent blinded outcome assessment was not feasible because this investigator-led trial was conducted within a single routine clinical service, where separate research personnel were not available to conduct blinded post-intervention assessments.

The flow of participants through recruitment, randomization, follow-up, and analysis is presented in the CONSORT flow diagram (Figure 1).

### 2.4. Intervention

The intervention was designed as a structured occupation-based group occupational therapy program, emphasizing the therapeutic use of meaningful occupations and functionally relevant activities, with cognitive exercises used as preparatory or supportive components rather than as the sole focus of the intervention. The program aimed to support cognitive functioning by embedding cognitive demands within everyday occupational performance and real-world problem-solving, social and community participation, and creative expression. This approach was informed by the Occupational Therapy Practice Framework and the concept of functional cognition, which emphasize the integration and use of cognitive abilities during everyday occupational performance [13,14].

The intervention lasted 12 weeks and consisted of two 90-min sessions per week, for a total of 24 sessions. The intervention protocol was developed before implementation by an occupational therapist with 15 years of clinical experience in adult mental health, who subsequently delivered all intervention sessions. Participants assigned to the intervention group were divided into four subgroups. The written protocol specified the objectives, session structure, therapeutic goals, core content, frequency, and duration of the intervention and was implemented consistently across all four subgroups. Minor adaptations were made only to the pace of delivery, task demands, level of support, and feedback provided, according to participants’ performance and support needs, without changing the central content or therapeutic aims of the activities. Attendance at each intervention session was recorded using session attendance logs. All participants assigned to the intervention group remained enrolled throughout the 12-week intervention period; detailed attendance data are reported in Section 3. However, no formal independent assessment of intervention fidelity was conducted.

Each session followed a consistent structure. The first part included a brief warm-up activity to support attention and facilitate engagement with the group. The main part included the principal therapeutic activity of the session, targeting cognitive functions and occupational performance demands through meaningful, structured activities. The final part included reflection and closure, during which participants were encouraged to identify the strategies they had used, discuss difficulties encountered, and consider how these strategies could be applied in everyday life.

The occupational therapist structured the therapeutic environment, provided instructions, graded task demands, offered verbal or practical support when needed, and encouraged self-monitoring, collaboration, and active participation. The group format also provided opportunities for interpersonal communication, shared problem-solving, the exchange of experiences, and cooperative engagement.

The intervention was delivered within the Psychiatric Department of the General Hospital of Argolida–Nursing Unit of Argos, in a room suitable for group therapeutic activities. The intervention group received the structured occupation-based group occupational therapy program in addition to treatment as usual, whereas the control group received treatment as usual alone. Participants in the control group did not participate in a structured occupational therapy program during the study period.

The intervention content was organized around four main focus areas: cognitive activation; everyday occupational performance and real-world problem-solving; social and community participation; and creative expression.

Cognitive activation activities included word games, recall tasks, categorization, anagrams, crosswords, visual attention exercises, spot-the-difference tasks, visuospatial activities, and word or movement sequences. These activities targeted attention, memory, processing speed, verbal fluency, semantic organization, visuospatial organization, executive functioning, and self-monitoring. Rather than being delivered as isolated cognitive drills, these activities were integrated into the therapeutic theme of each session and linked to meaningful occupations, everyday occupational performance, social participation, creative expression, or real-world problem-solving.

Activities addressing everyday occupational performance and real-world problem-solving focused on the organization and sequencing of everyday tasks, daily routine planning, shopping and meal planning, appointment and time management, community mobility and transportation, financial management, and problem-solving in common daily situations. These activities incorporated cognitive demands involving working memory, planning, sequencing, decision-making, cognitive flexibility, problem-solving, and self-monitoring. In two sessions, ChatGPT (GPT-5; OpenAI, San Francisco, CA, USA) was used as a therapist-guided external cognitive aid within occupation-based problem-solving, rather than as an autonomous therapist or stand-alone cognitive exercise. Participants first developed their own plans for anonymous everyday scenarios, including a community café outing. The occupational therapist then entered standardized prompts and displayed the responses on a shared screen for group evaluation and adaptation. Participants did not interact individually with ChatGPT and the same procedure was used across all four intervention subgroups. These activities were used to facilitate group analysis of everyday problems, identification of possible actions and consequences, evaluation of alternatives, and selection of practical strategies.

Social and community participation activities included pair and small-group work, group discussions, question-and-answer activities, presentations, role-playing, collaborative problem-solving, and emotion recognition and regulation. These activities addressed interpersonal communication, cooperation, collaborative decision-making, empathy, social problem-solving, and self-monitoring. A community-based activity involving participation in a creative writing workshop was also included to support the transfer and application of therapeutic strategies beyond the usual clinical group setting.

Creative expression activities included creative writing, story composition, storytelling using images or words, guided imagery, drawing, drama-based activities, imaginative object activities, nature-related activities, leisure planning, and participation in recreational activities. These activities provided opportunities for self-expression, verbal organization, imagination, emotional processing, social interaction, and engagement in meaningful leisure occupations.

Across all intervention focus areas, occupational therapy strategies such as task analysis, grading of task demands, verbal and practical support, feedback, reflection, strategy identification, and structured problem-solving were used to adapt task demands, support the use of cognitive strategies, and strengthen the connection between session activities and everyday occupational performance.

As an illustrative example of how the four intervention focus areas were integrated within a single session, a session focusing on shopping began with a theme-related verbal warm-up, during which participants generated words associated with shopping without repeating words previously mentioned by other group members. Additional preparatory activities included recalling and imagining shopping-related items, describing products without naming them, and using pantomime for other participants to identify selected products. The main activity involved planning and organizing a shopping task, including identifying necessary products, preparing a shopping list, sequencing the required steps, considering available time and financial constraints, and discussing possible difficulties and alternative solutions. During the closing phase, participants reflected on the cognitive and problem-solving strategies they had used and collaboratively created a group collage related to the session theme.

The main components of the intervention, indicative occupations and activities, and targeted cognitive functions and aspects of occupational performance are summarized in Table 1.

### 2.5. Instruments and Assessment Procedure

Cognitive functioning was assessed using complementary global and domain-specific measures, including the MMSE, ACE-III, M-ACE, CDT, and TMT Parts A and B.

Cognitive outcomes were assessed at baseline (T0) and immediately after completion of the 12-week intervention (T1), using the same standardized instruments at both time points. A demographic and clinical form developed for the study was used at baseline to collect information on participants’ age, sex, educational level, marital status, employment status, psychiatric diagnosis, duration of psychiatric follow-up, comorbid conditions, and medication adherence. These data were used to describe the study sample.

The Mini-Mental State Examination (MMSE) was used to assess global cognitive functioning. The MMSE evaluates orientation, registration, attention and calculation, recall, language, and visuoconstructive ability. Total scores range from 0 to 30, with higher scores indicating better cognitive functioning [20,21].

The Addenbrooke’s Cognitive Examination-III (ACE-III) was used to provide a broader assessment of cognitive functioning. The ACE-III evaluates five cognitive domains: attention and orientation, memory, verbal fluency, language, and visuospatial abilities. The total score ranges from 0 to 100, with higher scores indicating better cognitive performance [22,23]. The Mini-Addenbrooke’s Cognitive Examination (M-ACE) is a brief cognitive screening measure derived from the ACE-III. The M-ACE score was calculated from items included in the ACE-III administration and did not constitute a separate assessment. The total M-ACE score ranges from 0 to 30, with higher scores indicating better cognitive performance [23,24].

The Clock Drawing Test (CDT) was used as a brief measure involving visuospatial organization, planning, visuoconstructive ability, and executive functioning. Participants were asked to draw an analog clock, place the numbers in their correct positions, and set the hands to a specified time. Performance was evaluated using a four-point scoring system, with one point assigned for each of the following components: drawing a closed clock face, including all numbers from 1 to 12, arranging the numbers in the correct spatial positions, and correctly positioning the clock hands to indicate the requested time. Total scores ranged from 0 to 4, with higher scores indicating better performance [25].

The TMT Parts A and B were included from the outset of cognitive assessment and administered at both assessment time points. The TMT was used to assess processing speed, visual attention, cognitive flexibility, and executive functioning. The TMT consists of two parts. In Part A, participants were required to connect numbers in ascending order, primarily involving visual search, sustained attention, visuomotor speed, and processing speed. In Part B, participants alternated between numbers and letters in sequence, placing additional demands on cognitive flexibility, set-shifting, divided attention, and executive control. Performance was recorded as completion time in seconds, with longer completion times indicating poorer performance. Errors were also recorded where applicable [26,27].

Psychopathology, global functioning, health-related quality of life, and satisfaction with mental health services were assessed at T0 and T1 using the PANSS, GAF, SF-36, and VSQ-9, respectively. The PANSS comprises Positive, Negative, and General Psychopathology subscales, which are also combined to produce a total score; higher scores indicate greater symptom severity [28]. The GAF provides an overall rating of psychological, social, and occupational functioning, with higher scores indicating better functioning [29]. The SF-36 assesses eight health-related quality-of-life domains scored from 0 to 100, with higher scores indicating better perceived health [30]. The Visit-Specific Satisfaction Instrument (VSQ-9) assesses satisfaction with healthcare services, with higher scores indicating greater satisfaction [31].

All assessments took place within the Psychiatric Department of the General Hospital of Argolida–Nursing Unit of Argos, in a quiet and appropriately arranged room to ensure privacy, comfort, and concentration. The same assessment procedures were applied to both groups at both time points. Cognitive tests and clinical scales were administered by trained mental health professionals according to the standardized administration and scoring instructions of each instrument. Although outcome assessors were aware of participants’ group allocation, these standardized procedures were consistently applied across groups and assessment time points to promote measurement consistency.

### 2.6. Data Analysis

Quantitative variables were summarized using means and standard deviations (SDs), as well as medians and interquartile ranges (IQRs), while categorical variables were summarized using absolute and relative frequencies. Continuous variables were initially assessed for normality using the Kolmogorov–Smirnov test. For assessing the differences between the two groups in quantitative baseline characteristics standardized mean differences (SMDs) were calculated.

Repeated-measures analysis of variance (ANOVA) was used to evaluate changes from baseline to post-intervention in MMSE and CDT scores; TMT Parts A and B completion times and error counts; ACE-III total and domain scores; and M-ACE scores. The time × group interaction was examined to determine whether changes over time differed between the intervention and control groups. For the effect size of the time × group interaction, partial eta squared (η^2^) was reported.

Logarithmic transformations were applied when the assumption of normality was not satisfied, i.e., in all under study outcomes. In scales where zero was within score range, the transformation used was log(x + 1), where x is the score of the scale. Accordingly, *p* values for the time × group interaction were based on the transformed data where applicable, while descriptive statistics were presented on the original measurement scale. Bonferroni correction was applied in terms of sensitivity analysis.

The same analytic procedure, comprising normality assessment, logarithmic transformation where required, and repeated-measures ANOVA with the group × time interaction and partial η^2^, was applied to the registered secondary PANSS, GAF, SF-36, and VSQ-9 outcomes. The PANSS Total and SF-36 Emotional Well-Being scores satisfied the normality assumption and were therefore analysed on the original scale. Bonferroni correction across the 14 registered secondary outcome scores (α = 0.05/14 = 0.0036) was applied as a sensitivity analysis. Both unadjusted and Bonferroni-adjusted group × time *p* values for these outcomes are reported in Supplementary Tables S1 and S2.

All randomized participants completed the post-intervention assessment and were analyzed in the groups to which they had originally been allocated. There were no missing outcome data among the randomized participants; therefore, no imputation procedures were required. All reported *p* values were two-tailed, and statistical significance was set at *p* < 0.05. Statistical analyses were performed using IBM SPSS Statistics for Windows, version 27.0 (IBM Corp., Armonk, NY, USA).

## 3. Results

A total of 90 individuals were assessed for eligibility. Four were excluded before randomization because they did not meet the eligibility criteria. The remaining 86 participants were randomized, with 44 allocated to the intervention group and 42 to the control group. All randomized participants completed the post-intervention assessment and were included in the analysis. All 44 participants assigned to the intervention group remained enrolled throughout the 12-week intervention period. They attended a mean of 23.3 of the 24 scheduled sessions (SD = 0.8; range: 22–24), and 22 participants (50.0%) attended all 24 sessions. No adverse events, clinically significant deterioration, or withdrawals for clinical reasons were reported in either group during the study period. Baseline sociodemographic and clinical characteristics are presented in Table 2.

The mean age was 44.5 years (SD = 12.3) in the control group and 43.7 years (SD = 13.2) in the intervention group. Most participants were male in both groups (66.7% in the control group and 61.4% in the intervention group), had completed up to high school education (78.6% and 79.5%, respectively), and were unemployed (73.8% and 88.6%, respectively). Comorbid medical conditions were reported by 26.2% of participants in the control group and 13.6% in the intervention group, while a family history of psychiatric disorders was reported by 31.0% and 45.5%, respectively. Regular psychiatric follow-up was common in both groups, and approximately half of the participants had been followed for six years or longer (52.4% in the control group and 45.5% in the intervention group). At baseline, the mean PANSS Positive score was 17.36 (SD = 7.46) in the control group and 18.73 (SD = 7.28) in the intervention group (SMD = 0.19). The mean PANSS Negative score was 21.12 (SD = 5.61) and 22.55 (SD = 6.71), respectively (SMD = 0.23). The mean PANSS Positive–Negative Composite score was −3.76 (SD = 8.48) and −3.82 (SD = 7.99), respectively (SMD = 0.01), while the mean PANSS General Psychopathology score was 39.76 (SD = 10.86) and 44.34 (SD = 10.89), respectively (SMD = 0.42).

Mini-Mental State Examination (MMSE) and Clock Drawing Test (CDT) scores are presented in Table 3. At baseline, no significant between-group differences were observed for either MMSE or CDT scores (*p* > 0.05). Following the intervention, the intervention group demonstrated a significant improvement in MMSE scores (mean difference = 1.6; 95% CI: 1.13–2.06; *p* < 0.001), whereas no significant change was observed in the control group (*p* = 0.684). Similarly, CDT scores increased significantly in the intervention group (mean difference = 0.5; 95% CI: 0.3–0.8; *p* < 0.001), while no significant change was observed in the control group (*p* = 0.710). Since, in both scales the baseline scores were already high, the effect sizes of these differences were also calculated and found to be moderate to large (d = 1.04 for MMSE and d = 0.61 for CDT). At post-intervention, both MMSE (mean difference = 1.6; 95% CI: 0.9–2.3; *p* < 0.001) and CDT (mean difference = 0.3; 95% CI: 0.02–0.5; *p* = 0.045) scores were significantly higher in the intervention group compared with the control group. The group × time interaction was statistically significant for both outcomes, indicating a large effect for MMSE (*p* < 0.001, η^2^ = 0.19) and a medium effect for CDT performance (*p* = 0.018, η^2^ = 0.07).

Changes in ACE-III total and cognitive domain scores are presented in Table 4. No statistically significant between-group differences were observed at baseline for any of the ACE-III or M-ACE measures.

For ACE-III total score, both groups showed significant improvement over time. The control group increased from 82.9 (SD = 7.6) to 84.6 (SD = 6.7) (mean difference = 1.7; 95% CI: 0.6–2.9; *p* = 0.004), while the intervention group increased from 82.0 (SD = 8.1) to 90.5 (SD = 6.3) (mean difference = 8.5; 95% CI: 7.4–9.7; *p* < 0.001). The improvement was significantly greater in the intervention group, as indicated by a significant time × group interaction (*p* < 0.001, η^2^ = 0.43). The greater improvement in ACE-III total score in the intervention group is illustrated in Figure 2.

Regarding ACE-III cognitive domains, the intervention group showed significant improvements in attention, memory, verbal fluency, language, and visuospatial abilities (*p* < 0.001 for all within-group changes). In the control group, a significant improvement was observed only in memory (mean difference = 1.3; 95% CI: 0.5–2.0; *p* = 0.005). All time × group interactions were statistically significant, indicating greater improvements in the intervention group across ACE-III cognitive domains: attention (*p* = 0.033, η^2^ = 0.05), memory (*p* = 0.006, η^2^ = 0.09), verbal fluency (*p* = 0.009, η^2^ = 0.08), language (*p* < 0.001, η^2^ = 0.17), and visuospatial abilities (*p* < 0.001, η^2^ = 0.20).

Post-intervention between-group comparisons showed significantly higher scores in the intervention group for ACE-III total score (mean difference = 5.9; 95% CI: 3.1–8.7; *p* < 0.001), attention (mean difference = 0.7; 95% CI: 0.2–1.2; *p* = 0.012), verbal fluency (mean difference = 2.4; 95% CI: 1.5–3.2; *p* < 0.001), and visuospatial abilities (mean difference = 0.9; 95% CI: 0.2–1.5; *p* = 0.009). Differences in memory and language were not statistically significant.

Changes in Trail Making Test (TMT) completion time and errors as well as in Mini-Addenbrooke’s Cognitive Examination (M-ACE) are presented in Table 5. Lower completion times and fewer errors indicate better performance. No statistically significant between-group differences were observed at baseline or post-intervention for TMT Part A or Part B completion time or errors.

For TMT Part A completion time, the intervention group showed a significant reduction from 59.1 s (SD = 25.3) at baseline to 49.4 s (SD = 22.3) post-intervention (mean difference = −9.7; 95% CI: −13.4–−6.0; *p* < 0.001). The control group showed no significant change over time (*p* = 0.451). The time × group interaction was statistically significant, indicating a greater improvement in processing speed and visual attention in the intervention group (*p* < 0.001, η^2^ = 0.17). The change in TMT Part A completion time across the two assessment points is illustrated in Figure 3.

For TMT Part A errors, the intervention group showed a significant reduction over time (mean difference = −0.2; 95% CI: −0.4–−0.01; *p* = 0.015), whereas the control group showed no significant change (*p* = 0.701). The time × group interaction was also statistically significant, although the effect size was small (*p* = 0.048, η^2^ = 0.05).

For TMT Part B completion time, both groups showed significant reductions over time. The control group improved from 145.2 s (SD = 65.4) to 135.4 s (SD = 65.1) (mean difference = −9.8; 95% CI: −19.4–−0.3.; *p* = 0.016), while the intervention group improved from 160.7 s (SD = 80.5) to 129.8 s (SD = 60.0) (mean difference = −30.9; 95% CI: −40.4–−21.5; *p* < 0.001). The time × group interaction was statistically significant, indicating a greater improvement in the intervention group (*p* = 0.006, η^2^ = 0.09).

For TMT Part B errors, a significant reduction was observed in the intervention group (mean difference = −0.9; 95% CI: −1.4–−0.4; *p* = 0.004), whereas no significant change was observed in the control group (*p* = 0.873). However, the time × group interaction did not reach statistical significance (*p* = 0.056, η^2^ = 0.04).

M-ACE scores increased significantly in both groups. The control group improved from 22.8 (SD = 4.3) to 23.7 (SD = 3.5) (mean difference = 0.9; 95% CI: 0.1– 1.7; *p* = 0.013), while the intervention group improved from 22.0 (SD = 3.8) to 25.9 (SD = 3.2) (mean difference = +3.9; 95% CI: 3.3–4.6; *p* < 0.001). The time × group interaction was statistically significant, indicating a greater improvement in the intervention group (*p* < 0.001, η^2^ = 0.23). Post-intervention between-group comparisons showed significantly higher M-ACE score in the intervention group (mean difference = 2.2; 95% CI: 0.8–3.7; *p* = 0.004).

When Bonferroni correction was applied, in terms of sensitivity analysis, it was found that the results of primary outcomes concerning the degree of change in Mini Mental State Examination, the ACE-III total and its subscales “Language” and “Visuospatial abilities” remained significant, while the rest scales had no longer significant difference in the degree of change between the two groups. The results of the secondary outcomes for time of Trail Making Test Part A and for M-ACE total remained significant after Bonferroni correction, whereas the rest scores of Trail Making Test had no longer significant difference in the degree of change between the two groups.

The registered secondary clinical and functional outcomes are reported in Supplementary Tables S1 and S2. Following Bonferroni correction across the 14 outcomes, significant group × time effects remained for PANSS Positive (adjusted *p* = 0.011, partial η^2^ = 0.13), PANSS Negative (adjusted *p* < 0.001, partial η^2^ = 0.34), PANSS General Psychopathology (adjusted *p* < 0.001, partial η^2^ = 0.20), PANSS Total (adjusted *p* < 0.001, partial η^2^ = 0.19), GAF (adjusted *p* < 0.001, partial η^2^ = 0.56), and SF-36 Role Limitations–Physical Health (adjusted *p* < 0.001, partial η^2^ = 0.26). No other SF-36 domain or VSQ-9 group × time effect remained statistically significant after correction.

## 4. Discussion

The present randomized controlled trial examined the effects of adding a 12-week occupation-based group occupational therapy intervention to treatment as usual on cognitive test performance in adults with schizophrenia. Compared with treatment as usual alone, the addition of the intervention was associated with greater improvements in performance across several cognitive measures. After Bonferroni correction, significant group × time effects remained for the MMSE, ACE-III total score, M-ACE, TMT Part A completion time, and the ACE-III language and visuospatial domains. The effects observed for the remaining cognitive outcomes did not retain statistical significance after correction. Overall, the findings indicate improvements in selected measures of global and domain-specific cognitive test performance, rather than a uniform effect across all cognitive outcomes.

Examination of the individual cognitive test outcomes provides a more differentiated picture of the findings. The significant group × time effects for the ACE-III total score, M-ACE, and MMSE are consistent with broader improvements in global cognitive performance; however, because these measures overlap and the M-ACE is derived from the ACE-III, they should not be interpreted as independent confirmations of the intervention effect. In the unadjusted analyses, greater improvements were observed across all ACE-III cognitive domains; however, only the language and visuospatial domain effects remained statistically significant after Bonferroni correction. Similarly, both TMT Parts A and B completion times showed significant group × time effects in the unadjusted analyses, consistent with improved performance on tasks involving processing speed, visual search, divided attention, set-shifting, and cognitive flexibility [32,33]; however, only the TMT Part A completion-time effect remained statistically significant after correction. The group × time interaction for TMT Part B errors was not statistically significant.

Although the CDT showed a significant group × time effect in the unadjusted analysis, consistent with change on a multidimensional task involving visuospatial functioning, attention, memory, planning, and executive processes [34], this effect did not remain statistically significant after Bonferroni correction. The relatively high baseline MMSE values of approximately 27 and a baseline CDT median of 4, corresponding to the maximum possible score, indicate limited room for improvement and potential ceiling effects; changes in these measures should therefore be interpreted cautiously. Improvements in the control group on the ACE-III total score, M-ACE, ACE-III memory domain, and TMT Part B completion time may partly reflect practice or retest effects associated with repeated cognitive assessment [35]. Treatment as usual and other nonspecific influences may also have contributed, although these possibilities cannot be distinguished within the present study design. Although the magnitude of the observed group × time effects varied across outcomes, effect size alone does not establish clinical significance. Accordingly, the statistically significant group × time interactions should be interpreted as differential changes in performance on repeatedly administered cognitive tests and do not, by themselves, establish clinically meaningful cognitive improvement or transfer to everyday functioning. The findings that remained significant after correction provide the most robust evidence of differential change; nevertheless, because functional cognition, functional capacity, occupational performance, and real-world participation were not directly assessed, it remains uncertain whether these changes in cognitive test performance translated into meaningful changes in everyday functioning.

The registered secondary clinical, functional, and patient-reported outcomes presented in Supplementary Tables S1 and S2 showed greater improvements across several measures in the intervention group. After Bonferroni correction, significant group × time effects remained for the PANSS Positive, Negative, General Psychopathology, and total scores, GAF, and the SF-36 Role Limitations–Physical Health domain. These secondary findings should be interpreted cautiously because outcome assessors were aware of group allocation and several measures differed between groups at baseline. Moreover, the GAF and SF-36 do not constitute performance-based assessments of occupational performance or participation. Further studies with blinded outcome assessment are needed to confirm these findings.

The present findings can also be considered in relation to the broader evidence on cognitive rehabilitation and cognitive remediation in schizophrenia. Although the present intervention was not designed as a formal cognitive remediation program, evidence indicates that cognitive rehabilitation and remediation approaches can improve global cognition and several cognitive domains, including attention, memory, processing speed, and executive functioning [10,11,36,37]. Both computerized and non-computerized approaches have been associated with cognitive benefits, although the strength and methodological quality of the available evidence vary across intervention formats [36]. Evidence further indicates that cognitive remediation may be more effective when it includes the active involvement of a trained therapist, the structured development of cognitive strategies, and integration with broader psychiatric rehabilitation [11]. Moreover, techniques intended to facilitate the transfer of cognitive gains to real-world contexts, particularly when cognitive remediation is integrated with psychiatric rehabilitation, have been associated with more durable functional outcomes [12]. These elements partially overlap with the therapist-guided strategy support and functionally relevant activities incorporated into the present intervention; however, the two approaches should not be regarded as equivalent.

Within occupational therapy specifically, a growing, although still heterogeneous, body of research suggests potential cognitive and functional benefits from individualized, metacognitive, task-oriented, and occupation-focused interventions for people with schizophrenia. A multicenter randomized controlled trial found that the addition of individualized occupational therapy to usual group occupational therapy resulted in improvements across several cognitive domains and in overall cognitive functioning [15]. More recently, a randomized controlled trial combining computerized cognitive remediation with individualized occupational therapy reported improvements in global cognition, several cognitive domains, social functioning, and intrinsic motivation, although significant improvements in daily functioning and quality of life were not observed [38]. Other occupational therapy interventions have emphasized the application of cognitive and metacognitive strategies within everyday activities [39], task-oriented training involving real-world instrumental activities of daily living [40], and occupation-focused approaches designed to promote functional cognition, daily functioning, and participation [41]. A recent systematic review similarly identified promising effects of occupational therapy interventions on cognitive functioning in people with schizophrenia, while emphasizing substantial heterogeneity in study design, intervention content, intensity, and outcome measurement [16].

One possible explanation for the observed improvements in cognitive test performance is that cognitive demands were not limited to isolated exercises but were also embedded within meaningful and functionally relevant activities. These activities were designed to engage attention, memory, sequencing, planning, cognitive flexibility, and self-monitoring, while graded task demands and the group format may have supported strategy use, feedback, cooperation, and shared problem-solving. This approach is consistent with the concept of functional cognition, which emphasizes the integration and application of cognitive and performance abilities in everyday activity, and with the occupational therapy focus on engagement in meaningful occupations and the application of skills in daily life [13,14]. Functional cognition informed the rationale and content of the intervention but was not directly assessed as an outcome. Repeated engagement in progressively demanding activities may also have supported practice-based learning, the development of cognitive strategies, and their application across different intervention activities. More broadly, repeated cognitive practice has been discussed in the cognitive remediation literature in relation to experience-dependent learning and neuroplasticity [42]. However, no neural or biological mechanisms were assessed in the present study; therefore, any interpretation involving neuroplastic processes remains theoretical.

Clinically, the findings suggest the potential value of occupation-based group occupational therapy as an adjunct to usual care for adults with schizophrenia. The intervention was delivered within a routine mental health service and combined cognitive activation with everyday task practice, real-world problem-solving, social interaction, and creative activities in a group format. This clinical perspective is relevant because cognitive impairment in schizophrenia is closely associated with functional and rehabilitation outcomes [10].

From an occupational therapy perspective, cognitive difficulties may restrict engagement in and performance of meaningful everyday activities, as cognitive functioning has been associated with occupational engagement and the ability to perform daily occupations among people with schizophrenia [43,44]. The functional relevance of cognition is also evident in complex community-based occupations, such as driving, in which attention, executive functioning, processing speed, and visuospatial abilities have been associated with different aspects of driving performance and competence in people with schizophrenia [45]. This example reinforces the broader occupational therapy perspective that cognitive functioning should be interpreted in relation to the demands of meaningful everyday activities rather than solely as performance on isolated neuropsychological tests. Previous research further suggests that cognitive interventions may have greater functional relevance when cognitive strategy development is integrated with broader rehabilitation and applied within everyday or ecologically meaningful activities [11,12,40,41]. Accordingly, the intervention should be understood as an adjunct to, rather than an alternative to, established pharmacological and psychosocial care. The findings suggest that structured occupation-based group programs may complement existing treatment by embedding cognitive demands within activities relevant to everyday functioning and participation. However, because functional cognition, functional capacity, occupational performance, and real-world participation were not directly assessed, the present findings do not establish that improvements in cognitive test performance transferred to everyday occupational functioning.

The study has several strengths. Its randomized controlled design, concealed allocation, and complete post-intervention assessment of all randomized participants strengthen the internal validity of the findings. The intervention was delivered according to a predefined, structured 12-week protocol comprising 24 sessions, with attendance systematically recorded and all participants in the intervention group remaining enrolled throughout the intervention period. The use of the same experienced occupational therapist across all four subgroups supported consistency in intervention delivery.

A further strength is the clearly defined occupation-based orientation of the intervention, in which cognitive demands were embedded within meaningful occupations, everyday problem-solving, social participation, and creative activities rather than delivered solely as isolated cognitive exercises. Implementation within a routine psychiatric service, alongside treatment as usual, enhances the clinical relevance and potential feasibility of the intervention in real-world mental health care. In addition, the use of both global and domain-specific cognitive measures enabled a multidimensional examination of cognitive performance, while the inclusion of a treatment-as-usual control group enabled comparison of changes over time between the two groups.

Several limitations should be considered when interpreting the findings. First, the study was conducted in a single clinical setting, which may limit the generalizability of the results to other services, populations, and healthcare contexts. Because of the nature of the intervention, blinding of participants and the occupational therapist delivering the program was not feasible. In addition, outcome assessors were aware of group allocation. Although standardized administration and scoring procedures were consistently applied across groups and assessment time points, the absence of blinded outcome assessment may have increased the risk of detection bias. Because the intervention was delivered by a single occupational therapist, therapist-specific or allegiance effects cannot be excluded, and the generalizability of the findings to delivery by other therapists may be limited. Attendance was recorded and the intervention followed a predefined session structure; however, no formal independent treatment-fidelity assessment or session audit was conducted. Consequently, adherence to the intervention protocol was not independently verified.

The use of treatment as usual rather than an active, attention-matched control condition means that nonspecific effects associated with therapist contact, group participation, social interaction, and treatment expectancy cannot be excluded. Outcomes were assessed only immediately after completion of the intervention, without medium- or long-term follow-up. Therefore, the maintenance and durability of the observed cognitive improvements remain unknown. Furthermore, repeated administration of the same cognitive measures may have contributed to practice-related improvements in both groups.

Clinical stability was determined by the treating psychiatrists rather than through a prespecified PANSS severity threshold. Although baseline PANSS scores were available to describe psychopathology severity, data on illness duration, previous psychiatric hospitalizations, antipsychotic medication type, and chlorpromazine-equivalent dosage were not systematically collected. Duration of psychiatric follow-up should not be considered equivalent to duration of illness. The absence of these variables may have resulted in residual clinical confounding.

A schizophrenia-specific cognitive battery was not included. Instead, the study employed a combination of brief, clinically feasible cognitive measures assessing global cognition and selected cognitive domains. Although this approach facilitated assessment within routine mental health services, it may have provided less comprehensive coverage of schizophrenia-related cognitive impairment than a dedicated standardized battery. The MMSE, ACE-III, and M-ACE were administered to provide complementary levels of cognitive assessment, ranging from brief global screening to broader multidomain evaluation. However, because the M-ACE is derived from the ACE-III and all three measures assess overlapping aspects of global cognition, their findings should not be interpreted as fully independent confirmations of the intervention effect.

Moreover, because the cognitive outcome domain was assessed using three related co-primary measures and several secondary measures, individual findings should be interpreted cautiously. A Bonferroni-adjusted sensitivity analysis was conducted to address multiplicity; however, the overlap among measures should still be considered, particularly when interpreting secondary outcomes. Given the restricted, ordinal-like scoring of the CDT and the sparse distribution of TMT error counts, including numerous zero values, findings for these outcomes should be interpreted cautiously. A potential limitation of this study is the high baseline performance on the MMSE and CDT, which posed a risk of ceiling effects. Nonetheless, both measures demonstrated sensitivity to change, yielding effect sizes of *d* = 1.04 and *d* = 0.61, respectively. While these findings indicate that the instruments successfully captured the observed changes, they do not exclude the possibility of top-end score compression. Future studies involving cohorts with relatively high baseline cognitive scores would benefit from incorporating more granular cognitive batteries to further mitigate this limitation.

Functional cognition, functional capacity, occupational performance, and real-world participation were not directly assessed using occupation-based or performance-based outcome measures. Therefore, although the intervention incorporated functionally relevant and meaningful occupations, the observed improvements in cognitive test performance cannot be assumed to reflect transfer to everyday occupational functioning or participation.

Finally, the trial was retrospectively registered, which limits the independent verification of the prospective specification of outcomes and analyses. Although the study protocol, eligibility criteria, intervention procedures, and outcome measures had been defined before participant enrolment and no important changes were made after enrolment commenced, prospective registration would have strengthened transparency and reduced potential concerns regarding selective outcome reporting.

Future research should evaluate occupation-based occupational therapy interventions in larger and more diverse samples through multicenter randomized controlled trials incorporating active control conditions and, where feasible, blinded outcome assessment. Longer-term follow-up is needed to determine whether improvements in cognitive test performance are sustained and translate into meaningful changes in everyday occupational performance, social participation, and quality of life. Future studies should also include performance-based measures of occupational functioning, detailed reporting of participant-level adherence, independent assessment of intervention fidelity, and, where feasible, neuroimaging or neurophysiological measures to examine potential mechanisms of change.

## 5. Conclusions

In conclusion, this randomized controlled trial suggests that adding a 12-week occupation-based group occupational therapy intervention to treatment as usual may improve cognitive test performance in adults with schizophrenia. Compared with treatment as usual alone, the intervention was associated with greater improvements across several measures of global and domain-specific cognitive performance, although not all group × time effects remained statistically significant in the Bonferroni-adjusted sensitivity analysis. The clinical and long-term functional significance of these changes remains to be established. These findings should not be interpreted as evidence of improved everyday occupational functioning or participation, as these outcomes were not directly assessed. The results should also be interpreted cautiously given the retrospective trial registration, unblinded outcome assessment, and the use of multiple co-primary outcomes rather than a single prospectively specified primary endpoint. Taken together, the findings indicate that embedding cognitive demands within meaningful, structured, and occupation-based group activities warrants further evaluation as an adjunct to treatment as usual within rehabilitation-oriented care for adults with schizophrenia.

## Figures and Tables

**Figure 1:**
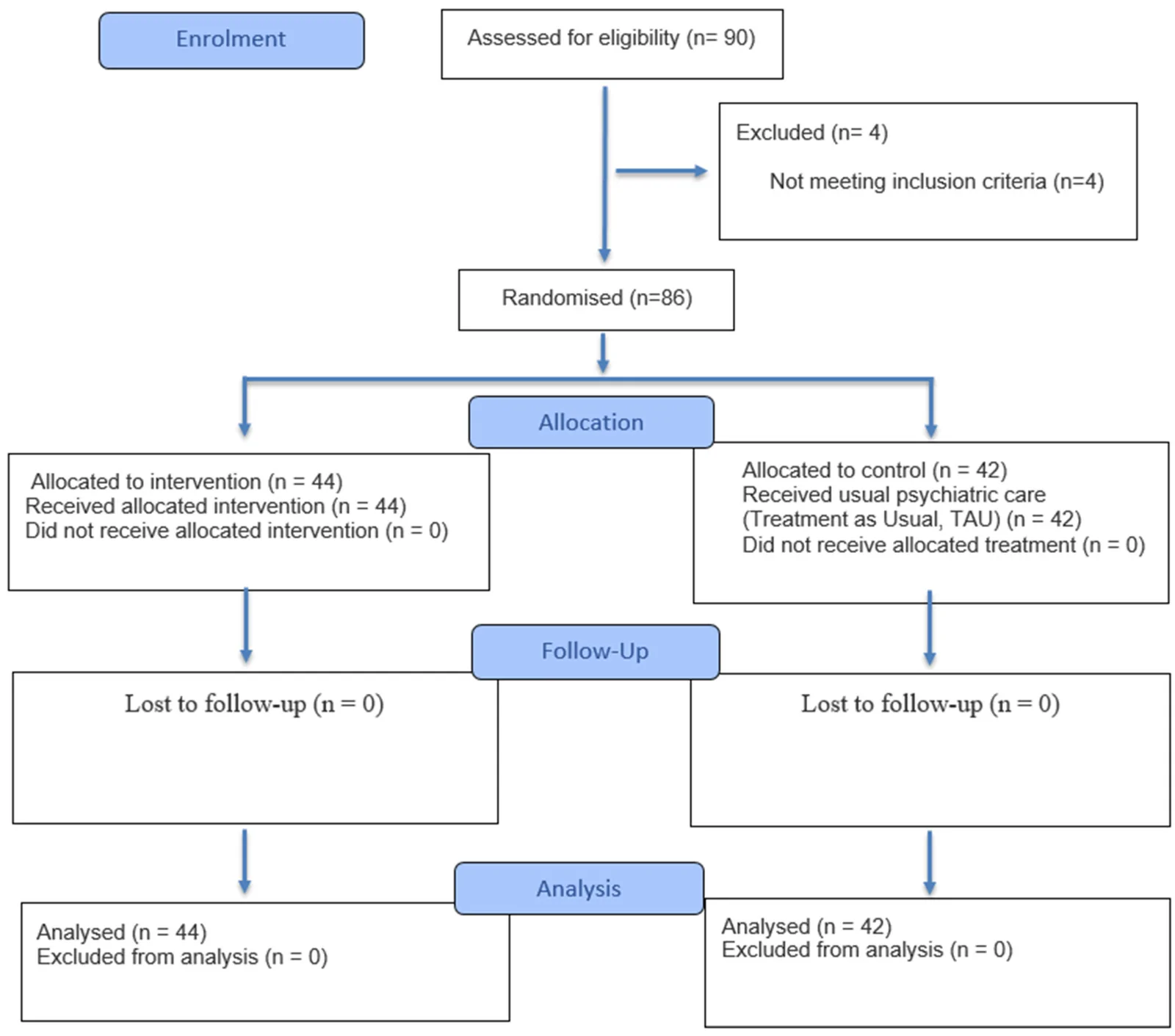
CONSORT 2025 flow diagram of participant progress through the trial.

**Figure 2:**
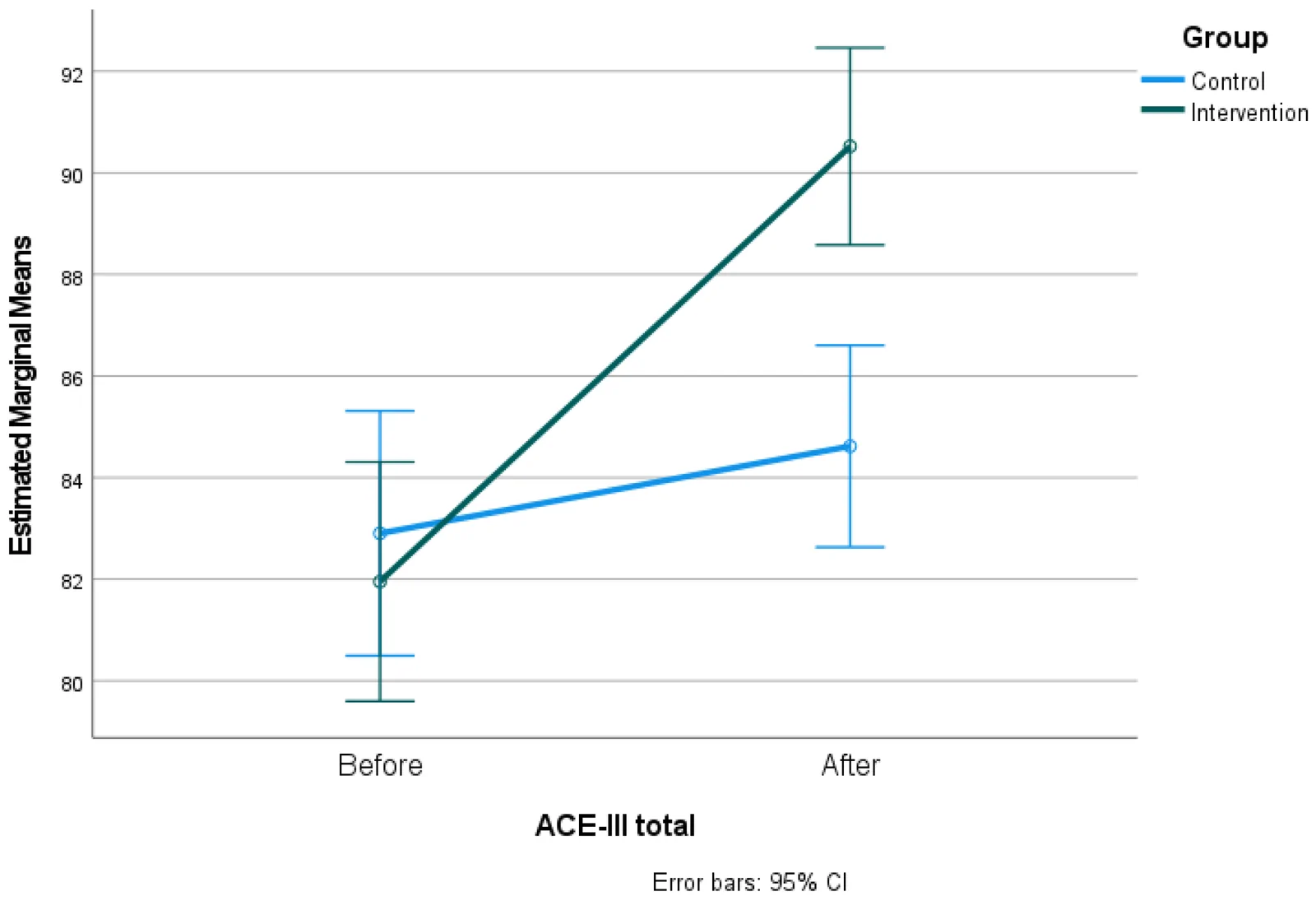
Change in Addenbrooke’s Cognitive Examination-III total score from baseline to post-intervention in the intervention and control groups. Error bars represent 95% confidence intervals.

**Figure 3:**
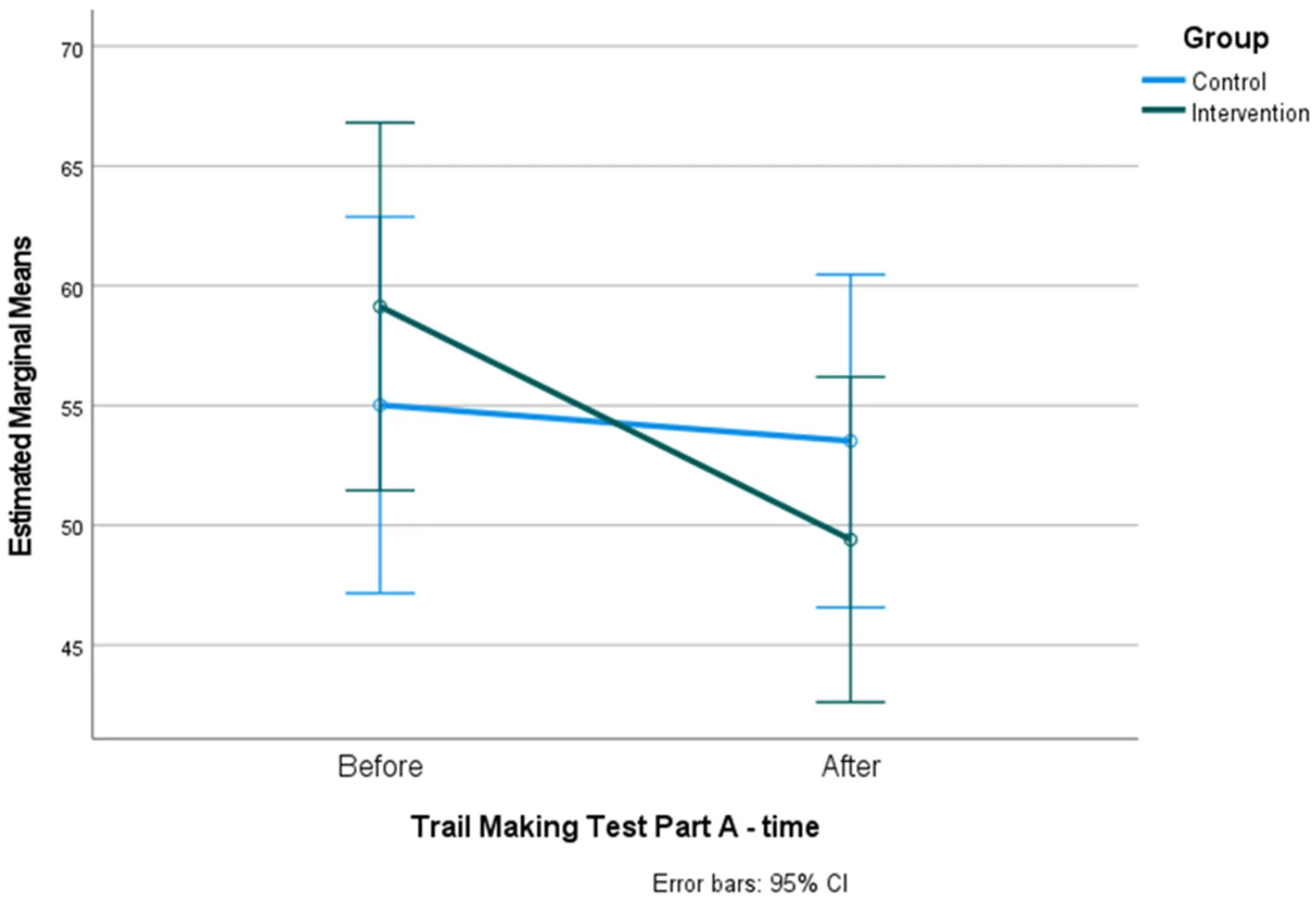
Change in Trail Making Test Part A completion time from baseline to post-intervention in the intervention and control groups. Error bars represent 95% confidence intervals.

**Table 1:** Overview of the occupation-based group occupational therapy intervention.

Occupational Therapy Intervention Focus	Indicative Activities and Occupations	Targeted Cognitive Functions and Aspects of Occupational Performance
Cognitive activation	Word games, recall tasks, categorization, anagrams, crosswords, visual-attention and spot-the-difference tasks, visuospatial activities, and word or movement sequences.	Attention, memory, processing speed, verbal fluency, semantic organization, visuospatial organization, executive functioning, and self-monitoring.
Everyday occupational performance and real-world problem-solving	Daily routine planning; organization and sequencing of everyday tasks; shopping and meal planning; appointment and time management; community mobility and transportation; financial management; management of common everyday problems; and selected therapist-guided activities using artificial intelligence tools.	Planning, organization, sequencing, working memory, decision-making, problem-solving, cognitive flexibility, functional judgment, self-management, and application of cognitive strategies in everyday situations.
Social and community participation	Pair and small-group activities, group discussions, question-and-answer activities, presentations, role-playing, collaborative problem-solving, emotion-recognition and regulation activities, and participation in a community creative-writing workshop.	Interpersonal communication, cooperation, collaborative decision-making, empathy, emotion recognition and regulation, social problem-solving, verbal expression, cognitive flexibility, and self-monitoring.
Creative expression	Creative writing, story composition, imaginative-object activities, storytelling using images or words, drawing, drama-based activities, guided imagery, nature-related activities, leisure planning, and participation in recreational activities.	Verbal fluency and expression, memory retrieval, associative and creative thinking, organization and sequencing of ideas, cognitive flexibility, imagination, emotional processing, self-expression, social interaction, and engagement in meaningful leisure occupations.

**Table 2:** Sample’s sociodemographic and clinical characteristics.

		Group			
		Control		Intervention	
		*n*	%	*n*	%
Gender	Male	28	66.7	27	61.4
	Female	14	33.3	17	38.6
Age, Mean (SD)		44.5 (12.3)		43.7 (13.2)	
Educational level	Up to high school	33	78.6	35	79.5
	Bachelor’s degree or higher	9	21.4	9	20.5
Employment	No	31	73.8	39	88.6
	Yes	11	26.2	5	11.4
Comorbid medical conditions	No	31	73.8	38	86.4
	Yes	11	26.2	6	13.6
Family history of psychiatric disorders	No	29	69.0	24	54.5
	Yes	13	31.0	20	45.5
Regular psychiatric follow-up	No	1	2.4	7	15.9
	Yes	41	97.6	37	84.1
Duration of psychiatric follow-up	1 year	7	16.7	9	20.5
	2–5 years	13	31.0	15	34.1
	6+ years	22	52.4	20	45.5
PANSS Positive, Mean (SD)		17.36 (7.46)		18.73 (7.28)	
PANSS Negative, Mean (SD)		21.12 (5.61)		22.55 (6.71)	
PANSS Composite, Mean (SD)		−3.76 (8.48)		−3.82 (7.99)	
PANSS General Psychopathology, Mean (SD)		39.76 (10.86)		44.34 (10.89)	

**Table 3:** Changes in primary outcomes: Mini Mental State Examination and Clock Drawing test by group.

	Before		After		Mean Change (SD)	P^2^	P^3^ (η^2^)
	Mean (SD)	Median (IQR)	Mean (SD)	Median (IQR)			
Mini Mental State Examination							
Control group	27.1 (2.1)	28 (26–29)	27 (1.7)	27 (26–28)	−0.1 (1.9)	0.684	<0.001 (0.19)
Intervention group	27 (2.5)	28 (25–29)	28.6 (1.5)	29 (27–30)	+1.6 (1.5)	<0.001	
P^1^	0.730		<0.001				
Clock Drawing test							
Control group	3.5 (0.8)	4 (3–4)	3.5 (0.7)	4 (3–4)	0.0 (0.9)	0.710	0.018 (0.07)
Intervention group	3.3 (1.0)	4 (3–4)	3.8 (0.4)	4 (4–4)	+0.5 (0.9)	<0.001	
P^1^	0.202		0.045				

P^1^: *p*-value for the comparison between the control and intervention groups. P^2^: *p*-value for the before-after comparison. P^3^: *p*-value for the comparison of the degree of change between groups (after logarithmic transformation).

**Table 4:** Changes in ACE-III total and cognitive domain scores by group.

	Before		After		Mean Change (SD)	P^2^	P^3^ (η^2^)
	Mean (SD)	Median (IQR)	Mean (SD)	Median (IQR)			
ACE-III total							
Control group	82.9 (7.6)	83.5 (77–88)	84.6 (6.7)	85.5 (81–89)	+1.7 (3.6)	0.004	<0.001 (0.43)
Intervention group	82 (8.1)	83 (78–88)	90.5 (6.3)	92 (88.5–96)	+8.5 (3.8)	<0.001	
P^1^	0.570		<0.001				
Attention							
Control group	16.5 (1.6)	17 (15–18)	16.8 (1.4)	17 (16–18)	+0.3 (1.3)	0.114	0.033 (0.05)
Intervention group	16.6 (1.6)	17 (15–18)	17.5 (1)	18 (17–18)	+0.9 (1.3)	<0.001	
P^1^	0.854		0.012				
Memory							
Control group	20 (4.5)	21 (16–24)	21.3 (3.8)	22.5 (18–24)	+1.3 (2.5)	0.005	0.006 (0.09)
Intervention group	19.4 (4.5)	19 (16.5–23)	22.6 (3.5)	24 (21–25)	+3.2 (3.1)	<0.001	
P^1^	0.560		0.113				
Verbal fluency							
Control group	8.1 (2.6)	8 (7–10)	8 (2.1)	8 (7–10)	−0.1 (1.7)	0.301	0.009 (0.08)
Intervention group	8.8 (1.9)	9 (7–10)	10.4 (1.8)	11 (9–12)	+1.6 (1.4)	<0.001	
P^1^	0.082		<0.001				
Language							
Control group	24 (2)	24.5 (23–25)	24.2 (1.7)	24.5 (24–25)	+0.2 (1.3)	0.283	<0.001 (0.17)
Intervention group	23.4 (2.8)	24.5 (21.5–25)	24.9 (1.9)	26 (24.5–26)	+1.5 (1.5)	<0.001	
P^1^	0.215		0.119				
Visuospatial abilities							
Control group	14.3 (1.8)	15 (13–16)	14.2 (1.7)	15 (13–16)	−0.1 (1.4)	0.816	<0.001 (0.20)
Intervention group	13.7 (2.2)	14 (12.5–16)	15.1 (1.3)	16 (15–16)	+1.4 (1.5)	<0.001	
P^1^	0.139		0.009				

P^1^: *p*-value for the comparison between the control and intervention groups. P^2^: *p*-value for the before-after comparison. P^3^: *p*-value for the comparison of the degree of change between groups (after logarithmic transformation).

**Table 5:** Changes in secondary outcomes: Mini-Addenbrooke’s Cognitive Examination (M-ACE) and Trail Making Test (TMT) completion time and errors by group.

	Before		After		Mean Change (SD)	P^2^	P^3^ (η^2^)
	Mean (SD)	Median (IQR)	Mean (SD)	Median (IQR)			
Trail Making Test Part A—time							
Control group	55 (25.9)	50 (37–63)	53.5 (23)	49 (36–65)	−1.5 (10.6)	0.451	<0.001 (0.17)
Intervention group	59.1 (25.3)	53.5 (40–69.5)	49.4 (22.3)	43.5 (32–70)	−9.7 (12.2)	<0.001	
P^1^	0.366		0.294				
Trail Making Test Part A—Mistakes							
Control group	0 (0.2)	0 (0–0)	0.1 (0.3)	0 (0–0)	+0.1 (0.3)	0.701	0.048 (0.05)
Intervention group	0.2 (0.6)	0 (0–0)	0 (0.2)	0 (0–0)	−0.2 (0.6)	0.015	
P^1^	0.116		0.289				
Trail Making Test Part B—time							
Control group	145.2 (65.4)	131.5 (90–180)	135.4 (65.1)	118.5 (96–155)	−9.8 (30.6)	0.016	0.006 (0.09)
Intervention group	160.7 (80.5)	125 (100.5–239)	129.8 (60)	112.5 (86–188.5)	−30.9 (31.2)	<0.001	
P^1^	0.473		0.613				
Trail Making Test Part B—Mistakes							
Control group	1.4 (1.8)	0.5 (0–3)	1.2 (1.4)	1 (0–2)	−0.2 (1.9)	0.873	0.056 (0.04)
Intervention group	1.7 (1.7)	1 (0–3)	0.8 (1)	0 (0–1.5)	−0.9 (1.6)	0.004	
P^1^	0.326		0.173				
M-ACE total							
Control group	22.8 (4.3)	23 (21–26)	23.7 (3.5)	24.5 (22–26)	+0.9 (2.5)	0.013	<0.001 (0.23)
Intervention group	22 (3.8)	23 (20–25)	25.9 (3.2)	27 (24–29)	+3.9 (2.2)	<0.001	
P^1^	0.430		0.004				

P^1^: *p*-value for the comparison between the control and intervention groups. P^2^: *p*-value for the before-after comparison. P^3^: *p*-value for the comparison of the degree of change between groups (after logarithmic transformation).

## Data Availability

The data presented in this study are available from the corresponding author upon request. The data are not publicly available due to ethical and privacy restrictions related to clinical participant data. The study protocol submitted to and approved by the relevant ethics committees is available from the corresponding author upon reasonable request. A separate statistical analysis plan was not prepared.

## Supplementary Materials

The following supporting information can be downloaded at: https://www.mdpi.com/article/10.3390/neurolint18100182/s1, Figure S1: Change in Mini-Mental State Examination score for the intervention and control groups; Figure S2: Change in Clock Drawing Test score for the intervention and control groups; Figure S3: Change in Trail Making Test Part B completion time for the intervention and control groups; Table S1: Changes in psychopathology (PANSS) and global functioning (GAF) by group; Table S2: Changes in health-related quality of life (SF-36) and satisfaction with mental health services (VSQ-9) by group.

## Abbreviations

The following abbreviations are used in this manuscript:

ACE-III	Addenbrooke’s Cognitive Examination III
ANOVA	Analysis of variance
CDT	Clock Drawing Test
CONSORT	Consolidated Standards of Reporting Trials
ICD-10	International Classification of Diseases, Tenth Revision
IQR	Interquartile range
ISRCTN	International Standard Randomised Controlled Trial Number
M-ACE	Mini-Addenbrooke’s Cognitive Examination
MMSE	Mini-Mental State Examination
SD	Standard deviation
SPSS	Statistical Package for the Social Sciences
TMT	Trail Making Test
