# Supplementary material for: Effects of an Occupation-Based Group Occupational Therapy Intervention on Cognitive Functioning in Adults with Schizophrenia: A Randomized Controlled Trial
Source: Neurol Int. 2026 Sep 25;18(10):182. doi: 10.3390/neurolint18100182 (PMC13708567; doi:10.3390/neurolint18100182)
Supplement: Supplementary file 1 [file neurolint-18-00182-s001.zip › neurolint-4502118-supplementary.pdf]

Figure S1. Change in Mini-Mental State Examination score for intervention and control group.

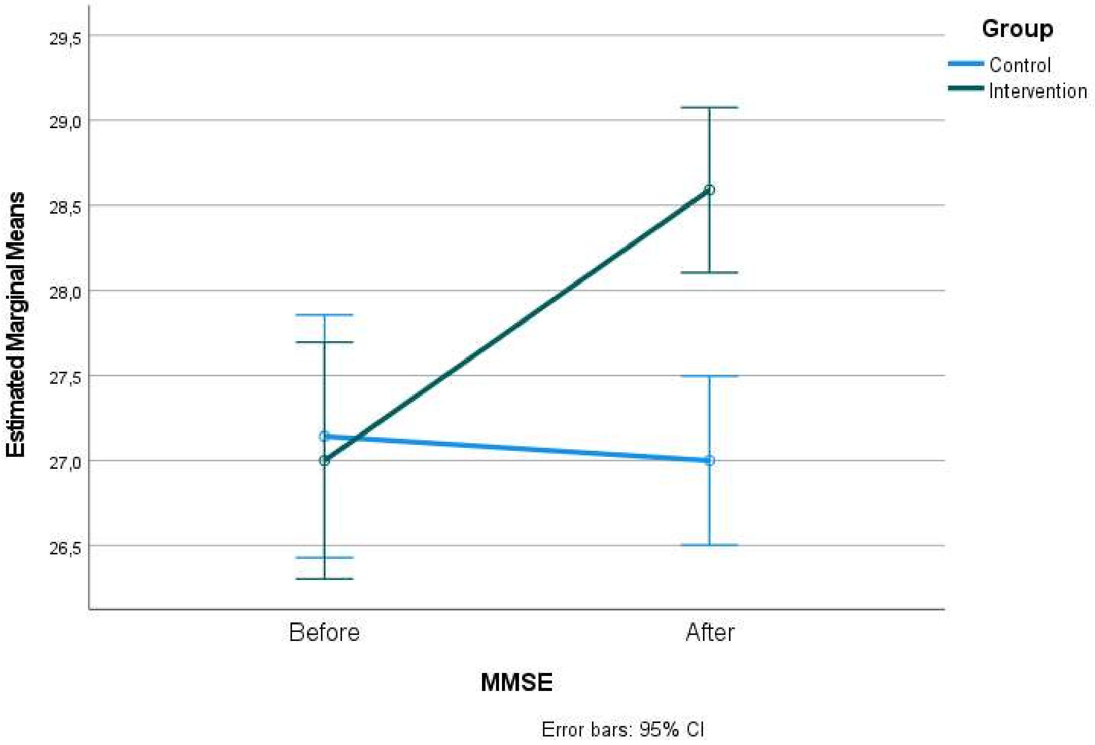

Figure S2. Change in Clock Drawing Test score for intervention and control group.

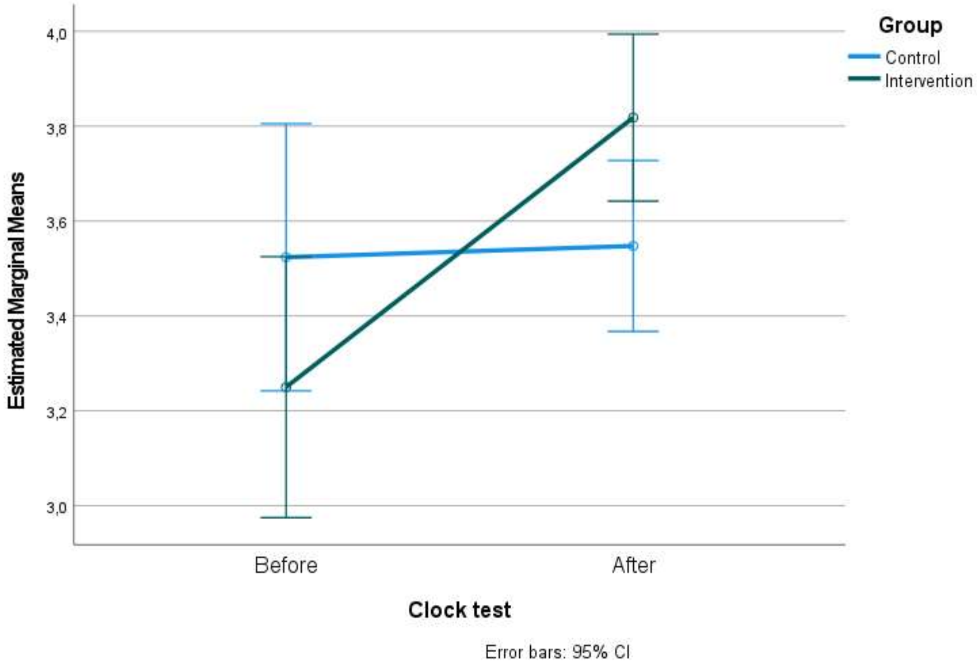

Figure S3. Change in Trail Making Test Part B completion time for intervention and control group.

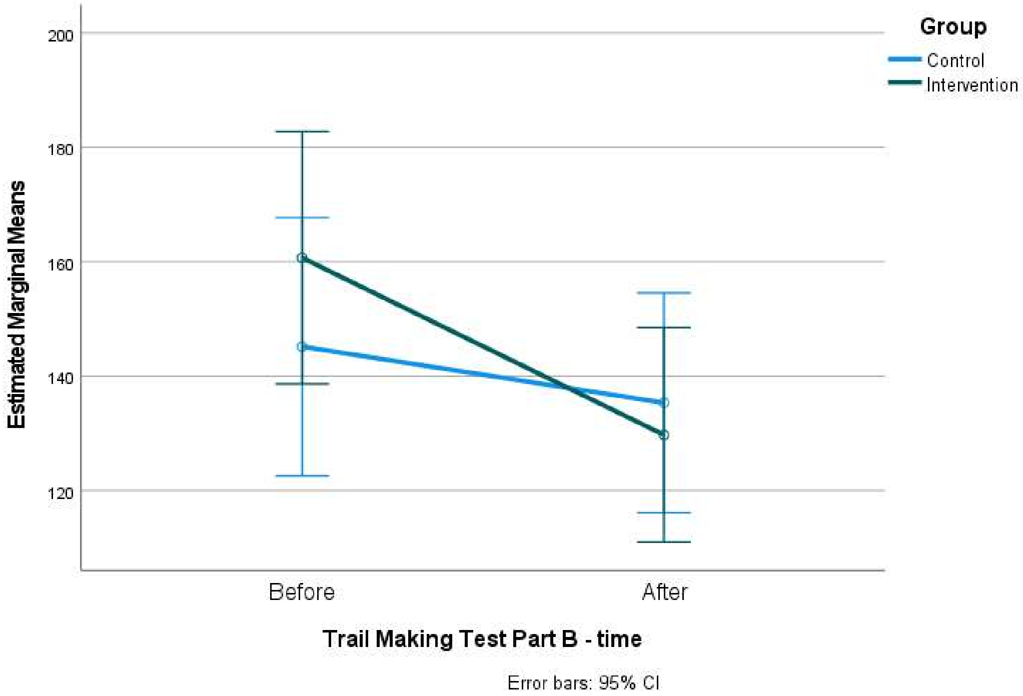

Table S1. Changes in psychopathology (PANSS) and global functioning (GAF) by group.

Outcome / Group	Before		After		Mean change (SD)	P2	P3	Partial η²	Bonferroni-adjusted P3
	Mean (SD)	Median (IQR)	Mean (SD)	Median (IQR)					
PANSS Positive									
Control group	17.4 (7.5)	16.5 (12–24)	14.8 (5.0)	14 (11–18)	−2.5 (4.9)	.008	<.001	0.13	.011
Intervention group	18.7 (7.3)	20 (12–23.5)	12.7 (4.2)	12 (10–16.5)	−6.1 (6.4)	<.001			
P1		.421		.045					
PANSS Negative									
Control group	21.1 (5.6)	20 (17–24)	20.5 (4.8)	19.5 (18–22)	−0.7 (3.2)	.335	<.001	0.34	<.001
Intervention group	22.5 (6.7)	22.5 (17.5–26.5)	17.7 (5.7)	18 (13.5–21)	−4.9 (3.3)	<.001			
P1		.440		.007					
PANSS General Psychopathology									
Control group	39.8 (10.9)	37 (34–42)	36.3 (7.9)	35 (32–39)	−3.4 (7.0)	.003	<.001	0.20	<.001
Intervention group	44.3 (10.9)	44 (36.5–49)	34.4 (8.4)	32 (28.5–41)	−9.9 (8.2)	<.001			
P1		.036		.210					
PANSS Total									
Control group	78.2 (19.9)	76.5 (65–85)	71.6 (13.5)	69 (63–79)	−6.6 (13.6)	.005	<.001	0.19	<.001
Intervention group	85.6 (21.3)	88 (72–97)	64.7 (15.9)	60.5 (56–75.5)	−20.9 (16.3)	<.001			
P1		.101		.033					
Global Assessment of Functioning (GAF)									
Control group	51.7 (4.7)	51 (50–55)	51.3 (4.4)	51 (50–55)	−0.4 (2.2)	.417	<.001	0.56	<.001
Intervention group	47.5 (6.2)	50 (41–51)	53.7 (4.6)	55 (50.5–56.5)	+6.2 (3.2)	<.001			
P1		<.001		.016					

P1: p-value for the comparison between the control and intervention groups at each time point.
P2: p-value for the before–after comparison within each group.
P3: p-value for the group × time interaction from the repeated-measures ANOVA; η^2: partial eta squared.
Bonferroni-adjusted P3: adjusted p-value for the group × time interaction across the 14 registered secondary outcome scores.
P1–P3 were calculated using log-transformed data, except for PANSS Total, which was normally distributed and analysed on the original scale. Descriptive statistics are presented on the original measurement scale.
Lower PANSS scores indicate lower symptom severity, whereas higher GAF scores indicate better functioning. PANSS, Positive and Negative Syndrome Scale; GAF, Global Assessment of Functioning; IQR, interquartile range

.

Table S2. Changes in health-related quality of life (SF-36) and satisfaction with services (VSQ-9) by group.

Outcome / Group	Before		After		Mean change (SD)	P2	P3	Partial η²	Bonferroni-adjusted P3
	Mean (SD)	Median (IQR)	Mean (SD)	Median (IQR)					
SF-36 Physical functioning									
Control group	76.5 (26.1)	90 (60–100)	74.4 (25.1)	82.5 (55–100)	−2.1 (12.6)	.828	.019	0.06	.261
Intervention group	64.3 (30.2)	75 (45–85)	72.3 (26.5)	80 (52.5–95)	+8.0 (23.1)	.002			
**P1**		.035		.639					
SF-36 Role limitations due to physical health									
Control group	60.7 (35.8)	62.5 (25–100)	57.1 (42.9)	62.5 (0–100)	−3.6 (32.9)	.110	<.001	0.26	<.001
Intervention group	29.0 (33.2)	25 (0–50)	63.6 (34.7)	75 (37.5–100)	+34.7 (31.5)	<.001			
**P1**		<.001		.121					
SF-36 Role limitations due to emotional problems									
Control group	65.1 (42.3)	100 (33.3–100)	55.6 (42.7)	66.7 (0–100)	−9.5 (35.5)	.352	.005	0.09	.065
Intervention group	42.4 (39.6)	33.3 (0–66.7)	65.9 (38.4)	66.7 (33.3–100)	+23.5 (40.4)	.002			
**P1**		.088		.163					
SF-36 Energy/fatigue									
Control group	56.9 (24.7)	55 (40–75)	57.6 (22.6)	57.5 (40–75)	+0.7 (17.8)	.572	.008	0.08	.111
Intervention group	46.9 (26.7)	50 (30–65)	60.1 (20.2)	60 (50–75)	+13.2 (15.0)	<.001			
**P1**		.057		.463					
SF-36 Emotional well-being									
Control group	61.3 (23.4)	64 (44–80)	64.7 (22.5)	68 (48–80)	+3.3 (14.8)	.162	.993	<0.01	1.000
Intervention group	59.6 (22.0)	58 (46–76)	63.0 (17.6)	64 (52–76)	+3.4 (15.8)	.149			
**P1**		.730		.702					
SF-36 Social functioning									
Control group	57.1 (29.2)	62.5 (37.5–87.5)	58.9 (28.2)	62.5 (37.5–75)	+1.8 (22.0)	.944	.006	0.09	.088
Intervention group	52.3 (28.6)	50 (37.5–75)	66.2 (22.0)	62.5 (50–81.3)	+13.9 (22.4)	<.001			
**P1**		.317		.068					
SF-36 Pain									
Control group	70.8 (29.7)	70 (55–100)	70.4 (28.5)	72.5 (45–100)	−0.4 (20.9)	.767	.050	0.04	.705
Intervention group	64.9 (32.6)	70 (36.3–100)	72.8 (26.3)	77.5 (45–100)	+7.8 (22.1)	.002			
**P1**		.400		.439					
SF-36 General health									
Control group	69.2 (20.1)	67.5 (55–85)	66.1 (24.7)	70 (45–90)	−3.1 (18.0)	.392	.007	0.08	.103
Intervention group	49.2 (22.8)	50 (35–65)	60.8 (20.2)	60 (50–70)	+11.6 (15.9)	.003			
**P1**		.011		.941					
VSQ-9 satisfaction with services									
Control group	83.2 (16.1)	86.1 (75–97.2)	85.1 (15.0)	88.9 (75–100)	+1.9 (8.6)	.385	.006	0.09	.081
Intervention group	75.3 (15.4)	75 (66.7–86.1)	86.2 (12.1)	88.9 (77.8–94.4)	+10.9 (16.9)	<.001			
**P1**		.034		.627					

P1: *p*-value for the comparison between the control and intervention groups at each time point.
P2: *p*-value for the before–after comparison within each group.
P3: *p*-value for the group × time interaction from the repeated-measures ANOVA; η^2: partial eta squared.
Bonferroni-adjusted P3 was calculated as the unadjusted group × time *p*-value multiplied by 14, capped at 1.
P1–P3 were calculated using log-transformed data, except for SF-36 Emotional Well-Being, which was normally distributed and analysed on the original scale. Descriptive statistics are presented on the original measurement scale. SF-36 domains were scored from 0 to 100 using the RAND 36-Item Health Survey 1.0 algorithm, and VSQ-9 scores were transformed to a 0–100 scale. Higher scores indicate better perceived health and greater satisfaction with services. SF-36, 36-Item Short Form Health Survey; VSQ-9, Visit-Specific Satisfaction Instrument; IQR, interquartile range.
